# Supplementary material for: Complexome profiling of the Chlamydomonas psb28 mutant reveals TEF5 as an early PSII assembly factor
Source: Plant Cell. 2025 Mar 17;37(6):koaf055. doi: 10.1093/plcell/koaf055 (PMC12164590; doi:10.1093/plcell/koaf055)
Supplement: koaf055_Supplementary_Data [file koaf055_supplementary_data.zip › psb28_interactive.zip!/plots/intensity/qualityPlots/peptides/HXK1_Cre02.g117500.t1.2.html]

### Cre02.g117500.t1.2

Trivial name: HXK1  
SeqCoverage [median]: 9.70  
Mass: 67.27 kDa  
  
 

| Sequence | MissedCleavage | Mass [Da] | wtIntensity | muIntensity | Score |
| --- | --- | --- | --- | --- | --- |
| AAAAAIMAAPAAAAAK | 0 | 1339.73 | 1172.38 | 2086.28 | 138.66 |
| AAMESALAAAK | 0 | 1032.53 | 1007.11 | 764.89 | 104.20 |
| LLSGMFLGETAR | 0 | 1293.68 | 1350.95 | 0.00 | 76.47 |
| RPLTDEQEAK | 1 | 1185.60 | 272.63 | 0.00 | 134.57 |
|  |  |  | 3803.1 | 2851.2 |  |
